# Death from Chloroform

**Published:** 1877-11

**Authors:** 


Death from Chloroform.—A patient who was about to submit to an operation for perineal fistula, died while under the effects of chloroform, recently, at the Blackburn Infirmary. There was nothing unusual in the case, and the post-mortem appearances were those of asphyxia. The case resembled many others in this, that death ensued quickly on the stage of struggling. We have often, when commenting on these cases, taken occasion to call attention to the danger which attends this stage of chloroform narcosis, when, owing to the rigid state of the muscles of respiration, the dangers of asphyxia are superadded to those which are inseparable from the use of chloroform. Whenever tonic muscular contraction ensues, the administrator should cease giving the anæsthetic, and watch the patient narrowly. The tonic muscular contraction is never of long duration, and its appearance is usually a sign that the patient, though unsteady, is, nevertheless, powerfully under the influence of the drug.—*London Lancet.*

